# Synergy-Dependent Center-of-Mass Control Strategies During Sit-to-Stand Movements

**DOI:** 10.1109/OJEMB.2024.3454970

**Published:** 2024-09-05

**Authors:** Simone Ranaldi, Leonardo Gizzi, Giacomo Severini, Cristiano De Marchis

**Affiliations:** ^1^ Department of Industrial, Electronics and Mechanical EngineeringRoma Tre University 19012 00154 Rome Italy; ^2^ Institute for Modelling and Simulation of Biomechanical SystemsUniversity of Stuttgart 9149 70174 Stuttgart Germany; ^3^ Fraunhofer Institute for Production Engineering and Automation, Department of Biomechatronics, Group Applied Biomechanics and Biosignal SensingUniversity of Stuttgart 9149 70174 Stuttgart Germany; ^4^ School of Electrical and Electronic EngineeringUniversity College Dublin 8797 4 Dublin Ireland; ^5^ Department of EngineeringUniversity of Messina 18980 98122 Messina Italy

**Keywords:** Muscle synergies, phase space analysis, sit-to-stand

## Abstract

The characterization, through the concept of muscle synergies, of clinical functional tests is a valid tool that has been widely adopted in the research field. While this theory has been exploited for a description of the motor control strategies underlying the biomechanical task, the biomechanical correlate of the synergistic activity is yet to be fully described. In this paper, the relationship between the activity of different synergies and the center of mass kinematic patterns has been investigated; in particular, a group of healthy subjects has been recruited to perform simple sit-to-stand tasks, and the electromyographic data has been recorded for the extraction of muscle synergies. An optimal model selection criterion has been adopted for dividing the participants by the number of synergies characterizing their own control schema. Synergistic activity has then been mapped onto the phase-space description of the center of mass kinematics, investigating whether a different number of synergies implies the exploration of different region of the phase-space itself. Results show how using an additional motor module allow for a wider trajectory in the phase-space, paving the way for the use of kinematic feedback to stimulate the activity of different synergies, with the aim of defining synergy-based rehabilitation or training protocols.

## I. Introduction

The sit-to-stand functional test is a widely adopted clinical tool for the characterization of biomechanical capabilities in the presence of different neurological pathologies [1], [2]. Considering the easiness of implementation of this test, and the importance of extracting the most information from relatively short experiments, several models have been developed in order to have a compact mathematical description of the interplay of the different biomechanical and neural variables in managing the task [3], [4]; most of those models are based on a phase-space representation of the lower body or centre-of-mass kinematics to unravel recurrent patterns and their functional variations [5], [6], [7].

The model proposed by Shinkoda and colleagues [5] is based on the joint description of the ankle, knee and hip angles, together with the centre-of-mass phase-space trajectory. This phase-space description explains the different subphases of the sit-to-stand movement and isolates features of the different biomechanical functions that are involved in this task. Other models exploit kinematics of the motion to build fuzzy controllers [6] or to populate the Hamilton equation of motion [7], all with the aim of identifying the different biomechanical functions that need to be achieved to perform a stable and safe sit-to-stand movement.

In addition to the kinematic and dynamic description of the sit-to-stand movement, several different studies have been focused on identifying, through electromyographic recordings, how the muscles control the movement during its different phases, especially under the framework of muscle synergy analysis [8], [9], [10], [11], [12], [13], [14]. The description through muscle synergies has been proven to be useful in identifying the causal part of the neuromechanical description of the sit-to-stand, determining the motor modules that are needed to achieve the patterns described by the kinematic laws that are reported in the literature.

In order to link the synergy-derived description of the movement with the kinematic results, it is critical to ensure that the methods involved for the synergy structure identification are optimal and unaffected by any processing or experimental choice [13], [15], [16], [17], [18]. Among all the quantities that are typically investigated through synergy analysis, the number of synergies represents the one with the most straightforward interpretation and the one that is mostly often used for the determination of the level of motor impairment in pathological movement [19], [20], [21].

In this paper, a group of healthy subjects has been recruited to perform simple sit-to-stand tests. Electromyographic data and centre-of-mass kinematics have then been analysed, in order to link the number of muscle synergies characterizing the control schema of each subject to the kinematic trajectories of the centre-of-mass in the sagittal plane. In detail, the CoM trajectory has been projected into the position-velocity phase space, both in cartesian and polar coordinates, and the synergy-to-kinematic mapping has been trained by considering the amplitude of the synergy activity as a function of the instantaneous position in the phase-space itself, showing how exploring different portions of this space requires the activation of different synergies. The choice of a bi-dimensional phase-space allows for the investigation of an invertible mapping, in which no states of the system are repeated more than one time during a sit-to-stand cycle; this invertible nature of the mapping might be a desirable feature of any system aiming to rehabilitate kinematics based on the synergies activity or to stimulate a specific synergy based on a kinematics-based biofeedback protocol.

## II. Results

### A. Synergy Number and Structure

Among the subjects that participated to the experiment, 7 were characterized by $N_{syn}=3$ and 5 by $N_{syn} = 4$. This number has been found to be independent from the anthropometric parameters.

**Figure 1.**
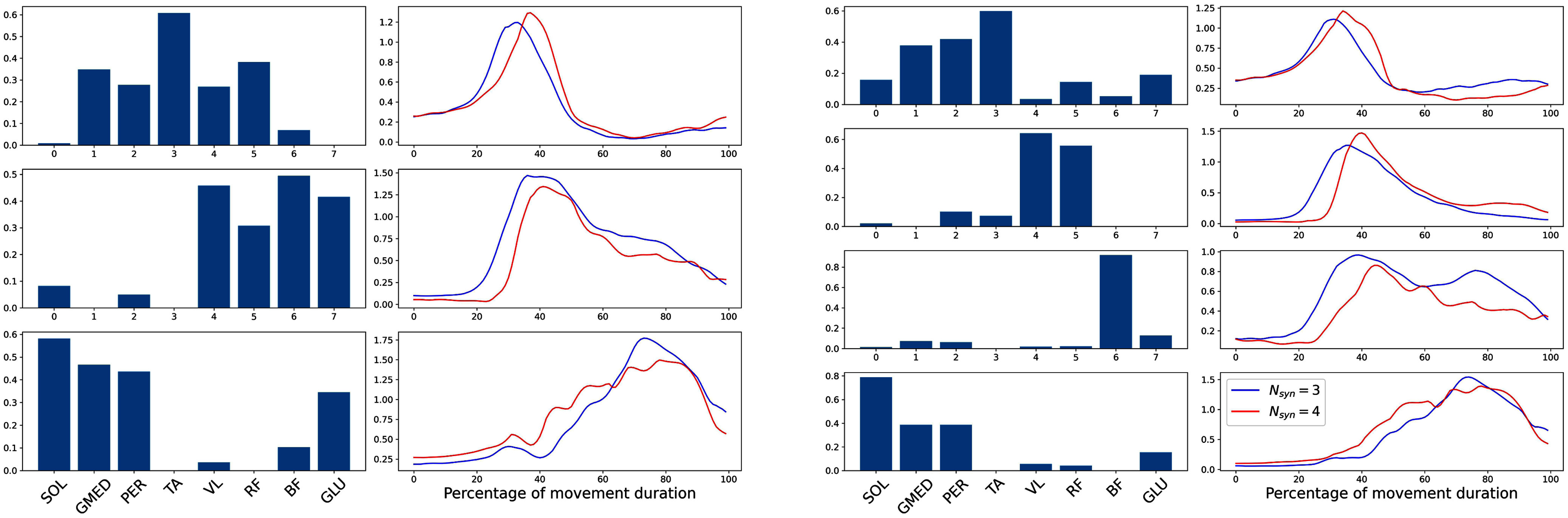
Synergy weights and activation coefficients for the two sub-groups. In blue the group with $N_{syn}=3$, in red the group with $N_{syn}=4$.

Concerning the structure of the synergies that are identified from the two groups (Fig. 1), the $W$ vectors show similarities in the modules that are active at the beginning and at the end of the movement, with differences between the two solutions in the central part of the cycle. The reconstructed activation coefficients show wider activations in the solution with $N_{syn} = 3$.

### B. Phase-Space Descriptions

**Figure 2.**
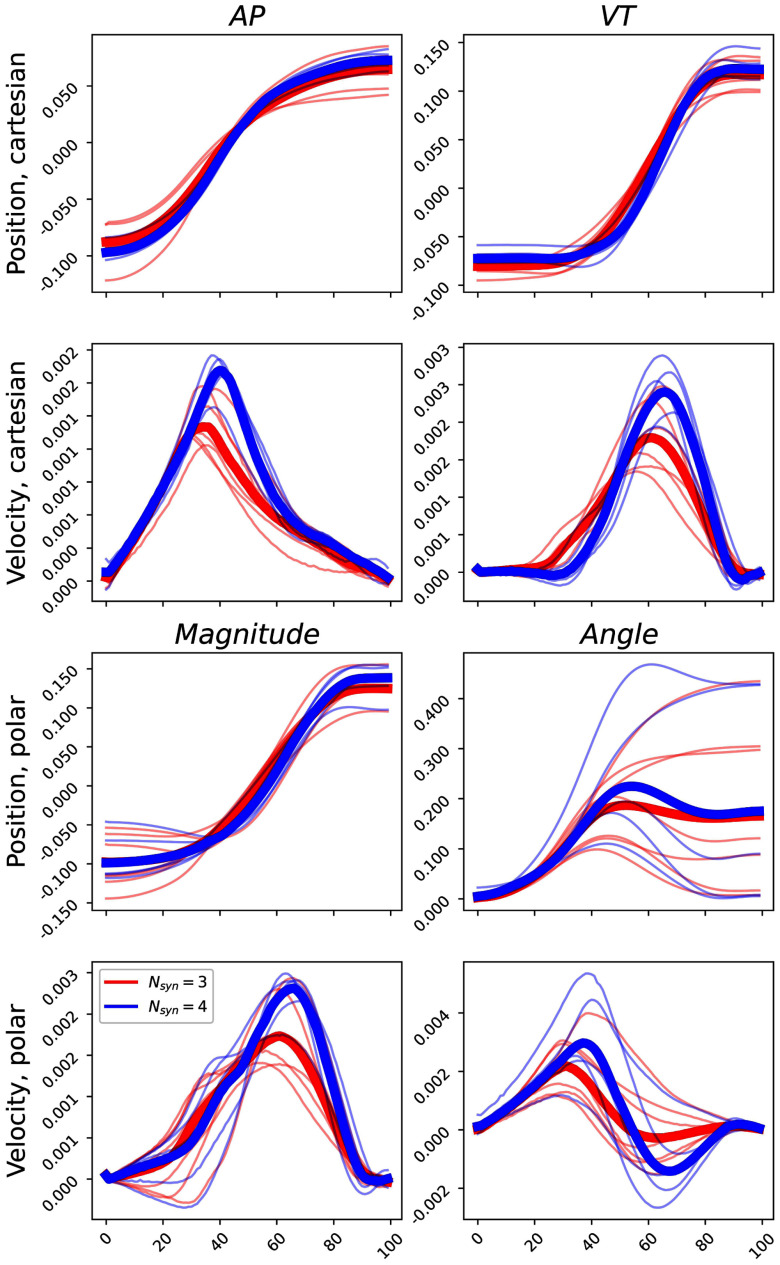
Average time course of the phase space variables, divided by the subject-specific $N_{syn}$. In blue the group with $N_{syn}=3$, in red the group with $N_{syn}=4$. All the values have been standardised prior to visualization.

The time course of the different phase-space variables that have been taken into account is shown in Fig. 2. Subjects with $N_{syn} = 4$ are characterized by generally higher peak velocity values, with no macroscopical differences in the trajectories, in both phase-space representation. Coherently with this results, the phase-space parameters that have been found to be significant are the vertical maximum velocity and its range variability (i.e. the standard deviation of the cycle-specific range).

**Figure 3.**
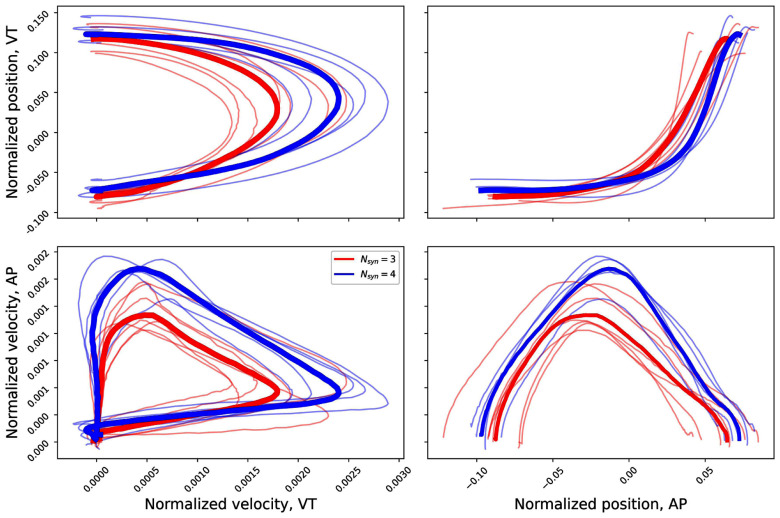
Position vs velocity plots for the cartesian representation of the sagittal plane. In blue the group with $N_{syn}=3$, in red the group with $N_{syn}=4$.

**Figure 4.**
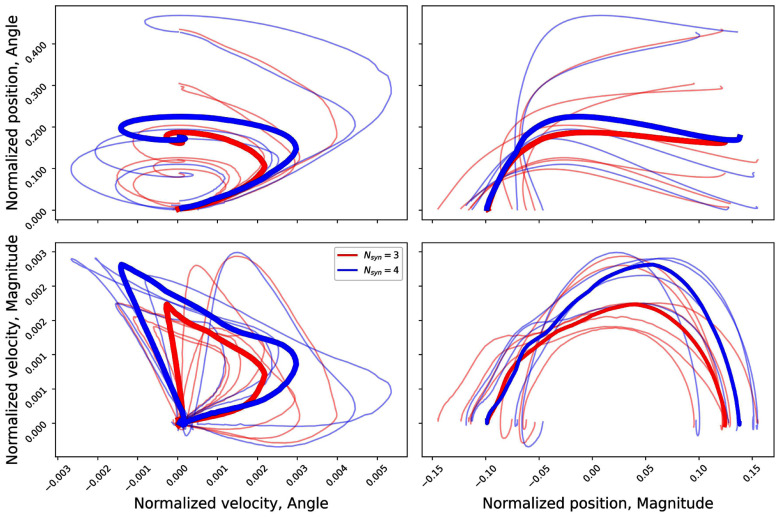
Position vs velocity plots for the polar representation of the sagittal plane. In blue the group with $N_{syn}=3$, in red the group with $N_{syn}=4$.

The same behaviour is more pronounced when looking at the two-dimensional representation in Figs. 3 and 4. The curves that the subjects with a higher number of synergies describe in all the spaces explore a wider (i.e. with a bigger area) region of the space itself.

### C. Synergy to Kinematics Mapping

**Figure 5.**
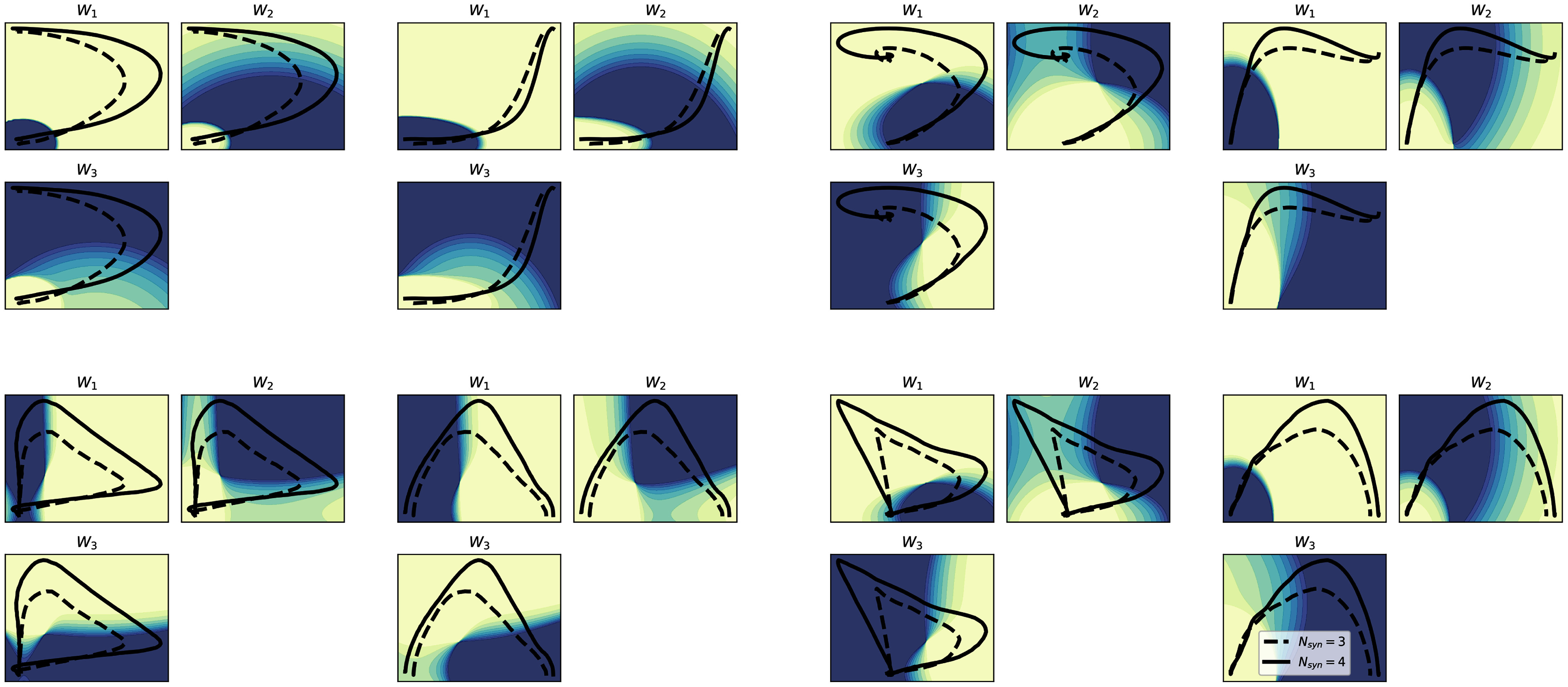
Mapping of the synergy activity on both phase-spaces for $N_{syn}=3$. The left half of the figure corresponds to the cartesian description, the right one to the polar. The curves, and their numerical values, are the same as presented in Figs. 3 and 4. For each phase space description, the three different plots correspond to the four different synergy activity mapping, a darker shade means a higher probability of that synergy being the most active among the three.

The mapping corresponding to $N_{syn}=3$ is shown in Fig. 5. With this representation, both the average phase-space curves corresponding to the two groups pass through the three distinct regions of the space. This mapping yielded a prediction accuracy on a test set composed of the 20% of the original data of at least 62%, with the highest values (higher than 70%) cartesian position-position, AP position-velocity and polar position-position combinations.

**Figure 6.**
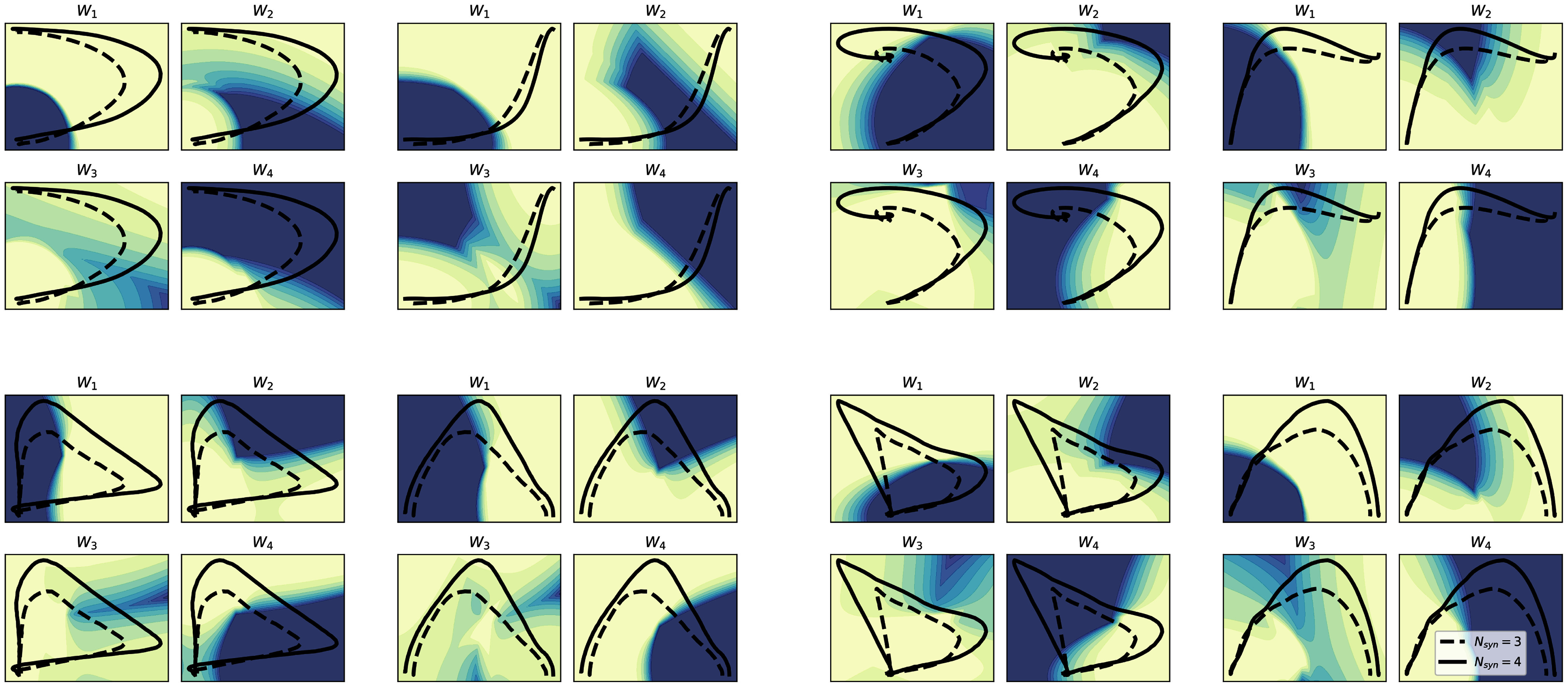
Mapping of the synergy activity on both phase-spaces for $N_{syn}=4$. The left half of the figure corresponds to the cartesian description, the right one to the polar. The curves, and their numerical values, are the same as presented in Figs. 3 and 4. For each phase space description, the four different plots correspond to the four different synergy activity mapping, a darker shade means a higher probability of that synergy being the most active among the four. In dashed the group with $N_{syn}=3$, in solid line the group with $N_{syn}=4$.

The results presented in Fig. 6 show that, when a subject is characterised by a 3-synergies coordination structure, the path in the phase-space does not generally pass through all the regions that are associated with a prominent activation of one specific synergies. Specifically, in the polar position-position and cartesian velocity-velocity representations only the subjects with 4 synergies pass, on average, in the region specific to the first synergy, while in the other representation, the subjects with a lower number of synergies tend to move closer to a superimposition region between different synergies.

**Figure 7.**
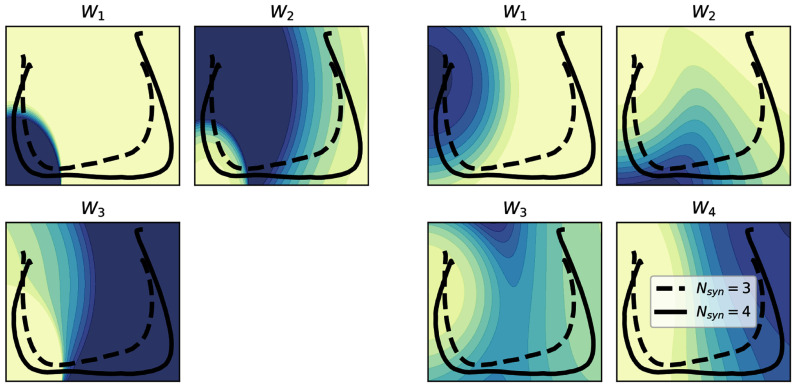
Mapping of the synergy activity on the 2D PCA space for both $N{syn}$ values. In dashed the group with $N_{syn}=3$, in solid line the group with $N_{syn}=4$.

Results from the PCA analysis are reported in Fig. 7. The 2D description given by the principal component analysis has been found to explain the 87% of the variance of the data. The mappings yielded a prediction accuracy higher than 70% for both the $N_{syn}$ values. From this description, it can be seen how the differences identified in the previous mappings are not reflected in a similar way, and how there is no synergy that identifies a region of space that is explored by only one of the two groups of subjects.

## III. Discussion

In this work, the sit to stand movement has been described by a compact phase-space description based solely on the trajectory of the subject centre-of-mass in the sagittal plane. While the commonly adopted phase-space description is based on joint angles [5], the description presented here is more compact, in the sense that it requires the tracking of one single point, thus being theoretically feasible using a single inertial sensor in an uncontrolled scenario. Even if the joint angle description yields more detail in the neuromuscular control strategies, with this description the aim is to build a model that can be tested and applied with an *ecological* experimental setup, thus increasing the feasability of real-time monitoring applications.

In this paper, the adoption of the objective $N_{syn}$ criterion developed in [15] has been made in order to increase the sensitivity to the presence of small-contribution motor modules such as $W_{3}$ in the solution with 4 synergies. While methods based on variance (i.e. $R^{2}$ or $VAF$ curve analysis) have a more widespread application in the current literature, such criterion are typically not able to identify such small changes, thus making the division between the two groups presented here less distinct.

The main focus of this analysis has been investigating the relationship between the muscle synergy model and the strategies with which the centre-of-mass kinematic is controlled during the sit-to-stand movement. In particular, the phase-space variables defined here have highlighted how the number of synergies involved in movement control is mainly affecting the management of the vertical direction, in both its position and velocity values during the cycle.

While the aforementioned result can reasonably being identified without the need of a phase-space description, the analysis of the trajectories is able to highlight more features of the neuromuscular control of this biomechanical task. In detail, the synergy to kinematic mapping proposed here has been able to show that the activity of different synergies is possibly arising by the necessity (by biomechanical constraints or by the adoption of different strategies) of exploring different points in the phase-space itself. The choice of using a 2D space, instead of each kinematic variable alone arises from the aim for an invertible mapping that can be used for rehabilitation purposes. More in detail, the phase space trajectory does not pass through the same point more than one time during one cycle; while some 1D variables have this characteristic, this is not true for the velocities and the angle coordinate, giving rise to the need of the 2D representation. The results coming from the PCA analysis, moreover, prove that this space has to be defined from kinematic variables and not from an unspecific representation.

The results coming from the mapping with $N_{syn}=3$ shows consistent results between the two groups of subjects, suggesting that the main regions of the phase-space can be explored thoroughly by the activity of only three synergies. However, the adoption of an objective criterion for the determination of the number of synergies has highlighted the presence of one additional module for a subset of the subjects; when this module is taken into account the regions of space that are explored highlight smaller portions that can be explained only by the activity of this additional module, and that are explored only by the subjects that show that additional module. In our description, we defined this mapping in a fuzzy manner, by using probability as the mapping output itself; in the solution with 4 synergies, as a consequence, the mapping is more ambiguous and most of the space is mapped as a superimposition of more than one synergy. This superposition, however is found to be present also in the temporal coefficients coming from the reconstruction (Fig. 1), supporting this choice as the optimal for the visualization of the relationship.

More in detail, the solution with $N_{syn} = 4$ is characterised by the split of the second synergy of the 3-modules solution. This split might be generated by the need of managing larger speed values, with the muscles that generate knee extension (i.e. the ones belonging to the second synergy) that are characterised by a more peaked activation. In this sense, it is reasonable to suppose that this separation of the synergies governing the central part of the movement is the result of a different strategy, in which the knee extension is pushed in a faster way, while the knee flexion (i.e. the activity of the third synergy) is prolonged and delayed to stabilize and decelerate the movement. This difference in the activation might be the explaining factor for the difference in the average centre-of-mass trajectories shown in Fig. 2.

In this analysis, all the mappings have been trained by using only two features, representing the two phase-space coordinates for each representation. With this choice, the presented results serve also as an indicator of which variables, and which description (phase-space, position-space or velocity-space) is controlled by the synergistic model. In this sense, the results seem to prove that most of the information in the centre-of-mass control by the synergies is encoded in the position in the cartesian coordinates (as per the $N_{syn}=3$ space); when the presence of the additional module is taken into account the focus is moved through a velocity-space, suggesting different mechanisms in which the additional synergy is needed for managing velocity adjustments and higher peak values.

The results of the phase-space description have a relatively high inter-subject variability that can hide some causal relationship between the number of synergy and the kinematic patterns, while still maintaining the information about any possible statistical correlation. The description presented here, however, shows a clear trend in the difference between the trajectories of the subjects adopting different control schemes, that gives rise to the need for a more detailed investigation of how those two features of the neuromechanical control are linked. It has to be stressed that in this work factors such as the anthropometric features have not be taken into account, so that further research is needed to test whether the presented behaviour is arising from a particular interplay between body dimensions and the mechanical constraints imposed by the specific task that has been realised. Moreover, the point of how this phase-space representation relates to the typical one based on joint angles is still open and might be the aim of subsequent research.

The results presented here are derived by a population of healthy people, thus being limited in the prediction of this relationship in the presence of a pathology. It is important to note, however, that, while the nature of this mapping might change in those cases, it is reasonable to suppose that such a mapping would still be present after a pathology. Considering this, a deeper investigation of this relationship can pave the way for the definition of advanced rehabilitation protocols, in which the phase-space trajectories are stimulated or imposed externally in order to aid or force the activity of one or more specific motor modules, aiming for a functionally targeted rehabilitation. Moreover, the kinematic parameters that have been used here can be easily recorded through wearable sensors [22], in the definition of a portable device for the realization of such rehabilitation protocols.

## IV. Conclusion

In this paper, a first evidence of a direct mapping between center-of-mass kinematics and muscle synergy activity in the case of different complexity levels of the motor control strategies has been given. Such a mapping has been extracted via the use of information-based and objective processing methods that allowed a fine characterization of the synergistic structure. The results show how a different number and structure of synergies can allow wider phase-space trajectories of the centre-of-mass; this description of the neuromechanics of the sit-to-stand movement can be highly useful in the determination of functional rehabilitation protocols that can be realised easily and controlled online via the simple tracking of the centre-of-mass kinematics.

## V. Materials and Methods

### A. Experimental Protocol

In total, 12 healthy subjects were involved in the study (age: $40 \pm 10$; height: $177 \pm 6$ cm; weight: $79 \pm 11$). The subject were asked to perform three trials of *30 seconds sit to stand* tests, with arms folded on the trunk and with the only instruction of trying to perform as many repetition of the movement as possible. According to [13], random cycles were selected for the analysis in order to have an equivalent total length of the trial that was consistent with the number of cycles in a *30-seconds sit to stand* task.

For the subsequent analysis, only the upward phase of a complete sit to stand to sit cycle has been considered, to avoid the influence of passive, gravity-driven strategies that might be involved in the return phases.

Kinematic has been recorded through a stereophotogrammetric system (BTS SMART-DX 6000, BTS Bioengineering, Milan, Italy), with a full body marker set, and surface EMG activity has been recorded at a sampling rate of 1 kHz through 8 wireless probes (BTS FREEEMG 1000, BTS Bioengineering, Milan, Italy), placed on the *soleus*, *gastrocnemius medialis*, *peroneus longus*, *tibialis anterior*, *vastus lateralis*, *rectus femoris*, *biceps femoris* and *gluteus medius* muscles of the right leg (corresponding to the dominant leg in the whole population under analysis).

### B. Computation of Kinematic Parameters

The center of mass of the subject's body has been estimated following a segmental method based on the anthropometric tables in [23]. Its trajectory has then been projected into the sagittal plane (defined by the vertical direction VT and the antero-posterior axis of the chair AP), and all the references have been expressed as the distance from the center of the base of support (i.e. the mid-point between the two *malleolus* markers).

Two different coordinates systems have been defined, one cartesian (AP, VT)

\begin{equation*}
x \qquad y \tag{1}
\end{equation*}

and one polar (Magnitude, Angle)

\begin{equation*}
\sqrt{x^{2}+y^{2}} \qquad \arctan {\frac{x}{y}} \tag{2}
\end{equation*}

these two coordinate systems have been then used for defining two different position-velocity phase spaces.

These two different coordinate systems represent two different possible strategies for kinematic control, one related to a VT vs AP set of coordinates (i.e. cartesian coordinates of the CoM) and one related to length and orientation of the vector representing the position of the body with respect to its approximate anchor point on the ground during upright stance (i.e. polar coordinates of the CoM).

In these phase spaces, parameters related to maximum and minimum values, as well as variability and ranges have been calculated for each of the four variables. Moreover, average time course for each subject has been calculated by resampling each sit to stand cycle over 100 samples.

### C. Synergy Related Parameters

Synergies have been extracted from the surface EMG envelope through Non-Negative Matrix Factorizaton [24]. The number of synergies $N_{syn}$ has been selected through the modified Akaike Information Criterion described in [15].

Subjects have been divided by their $N_{syn}$, and an average set of synergy vector $W$ has been defined for each $N_{syn}$, and then been used to reconstruct all the original muscle activations of all the subjects, regardless of their original number of synergies. The reconstructed profiles $H$ of the subjects which $N_{syn}$ was equal to the number of synergies of the reference vector have been used for subsequent analysis, while cross-reconstructed profiles (i.e. profiles reconstructed with a number of synergies different from the original) have been kept for visualization purposes.

### D. Kinematics to Synergy Mapping

The 8 phase-space variables, before the time-normalization and averaging have been used as features in input to a state to synergy mapping model. Mappings have been realised in a $N_{syn}$-dependent fashion, using the reconstructed synergy activation profiles $H$ and the features coming only from the subjects with a specific number of synergies.

Mappings have been realised in 2-dimensional spaces corresponding to the 4 combinations of variables in the cartesian representations and the 4 combinations for the polar description. In the specific, the mapping has been evaluated by training a Gaussian Naive Bayes classifier, with each combination of variables as input, and the index of the most active synergy as output. The probability of each synergy to be the most active in a specific point of the space is considered the output of the mapping, and used for further considerations.

A further mapping has been realised on the two dimensional description found by applying Principal Component Analysis to all the 8 phase-space variables and selecting the first two components (ranked by their percentage of explained variance).

### E. Statistical Analysis

The statistical influence of $N_{syn}$ on the phase-space parameters has been tested with a Kruskal-Wallis test, with the significance level set to 0.05.

*Conflict of Interest Statement:* The authors declare no conflict of interest.

*Author Contributions:* Writing - Original Draft: SR. Writing - Review and Editing: SR, LG, GS, CDM. Software: SR. Methodology: SR, CDM. Investigation: SR, CDM. Conceptualization: SR, LG, GS, CDM. Resources: CDM. Supervision: CDM. Project administration: CDM. Funding acquisition: LG, GS, CDM.

*Ethics Approval:* The study was approved by the Roma Tre University ethics committee.
